# Phage Therapy, Lysin Therapy, and Antibiotics: A Trio Due to Come

**DOI:** 10.3390/antibiotics9090604

**Published:** 2020-09-15

**Authors:** Tomás G. Villa, Carmen Sieiro

**Affiliations:** 1 Department of Microbiology and Parasitology, University of Santiago de Compostela, 15706 Santiago de Compostela, Spain; 2 Department of Functional Biology and Health Sciences, Area of Microbiology, University of Vigo, Lagoas-Marcosende, 36310 Vigo, Spain

Since their introduction, at the beginning of the 20th century, antibiotics were regarded as “magic-bullets”, a term coined by Paul Ehrlich, and, for several decades, considered as the universal panacea to combat pathogenic and/or undesirable bacteria. However, the genesis of spontaneous bacterial mutants, together with the overuse and misuse of these compounds, resulted in the emergence of antibiotic-resistant pathogens. In fact, these pathogenic bacteria often display multi-resistance to the antibiotics commonly used to treat the infections they produce. These multi-resistance bacteria include, not only hospital strains, but also microorganisms representing important human and animal pathogens, as well as those caused by plant diseases. The current situation is critical, as the extent of bacterial resistance is such that many diseases can no longer be effectively treated with the antibiotics available, with the consequent risk of returning to the conditions experienced during the “pre-antibiotic era”. In the current conjuncture, and taking into account the slow pace of discovery and/or development of new antibiotics, it is time to revive the concept of phage therapy, originally proposed by d’Herelle in the 1930s. Phages (bacteriophages) are viruses that specifically kill bacteria, which were discovered a hundred years ago. Phage therapy did not flourish in Western countries, due to the prominent role played by antibiotics; but both bacteriophages and the endolysins they produce have recently acquired prominence in the West, as alternative or combined therapy to treat antibiotic-resistant pathogens. The failures and mistakes of the past should teach us not to solely rely on the current antibiotic-based chemotherapy, but to design novel strategies that provide a two-pronged approach in the fight against pathogenic bacteria. Antibiotic treatment in combination with phage therapy provides a better control of undesirable bacteria, in particular of those microorganisms exhibiting multi-resistant phenotypes, as well as reducing the emergence of antimicrobial-resistant bacterial strains.

This Special Issue of “Phage Therapy, Lysin Therapy, and Antibiotics: A Trio Due to Come”, includes nine articles (four research publications and five reviews) with a focus on recent advances in these combination therapies.

The research articles included in this monograph concern the isolation of new antibiotic-resistant bacterial strains, as well as studying their susceptibility to either phages or the endolysins they produce. Other aspects involve the characterization and effectiveness of novel modified endolysins, identification of phage insensitive mutants, and the design of combination treatments capable of preventing the appearance of the mutants mentioned above. Accordingly, Salas et al. [1] isolated and characterized 77 methicillin-resistant *Staphylococcus aureus* strains and assessed their susceptibility to two phages and one endolysin. The authors reported that most of the strains were susceptible to phage phiIPLA-RODI, while all of the bacterial strains studied displayed some degree of susceptibility to the endolysin LysRODI. Salas et al. also described a correlation between susceptibility to endolysin and the presence of particular virulence genes, providing information that could help us understand the factors that affect the effectiveness of endolysins; this knowledge is essential to development of effective therapies against MRSA infections. The study by Abril et al. [2] addresses the importance of identifying the bacteria responsible for mastitis infections, as well as determining their antibiotic resistance, in order to formulate effective treatment therapies. These authors report the analysis and identification of *Streptococcus* peptides, isolated from dairy products, that act as either virulence factors, toxins, anti-toxins, provide resistance to antibiotics that are associated with the production of lantibiotic-related compounds, or play a role in the resistance to toxic substances. The study identified 134 peptides specific to *Streptococcus* spp., with two confirmed to be species-specific to *Streptococcus dysgalactiae.* In their conclusions, the authors raise the possibility of using endolysins and/or phages as a therapy in the treatment of mastitis-causing bacteria, in particularly when dealing with strains resistant to antibiotics. The article by Vazek et al. [3] describes two recombinantly modified antistaphylococcal enzymes, derived from either lysostaphin (LYSSTAPH-S) or endolysin (LYSDERM-S), that were obtained from kayvirus 812F1; these enzymes, in particular LYSSTAPH-S, are effective against a wide variety of methicillin-resistant *Staphylococcus aureus* (MRSA) strains. The authors propose that derivative enzymes could be further modified, with the aim of improving their antimicrobial properties and stability. Zhong et al. [4] isolated and characterized T4 bacteriophage insensitive mutants (BIMs) from *Escherichia coli*; a six-amino-acid deletion in gene *waaG* conferred phage resistance, by deactivating the addition of T4 receptor glucose to the lipopolysaccharide (LPS). The phage truncated the cellular LPS, which, in turn, destabilized the outer membrane and sensitized BIM to the food grade surfactant sodium dodecyl sulfate. This approach was used to design a T4-SDS combination which effectively prevented the generation of BIMs, resulting in the elimination of the bacteria.

This special monograph also includes a variety of review articles that analyze the development, evolution and current status of phage therapy, both in the clinical field and in agriculture and aquaculture. The review by de Miguel and co-workers [5] focuses on the major public health problem caused by the rapid appearance of antibiotic-resistant strains responsible for urinary tract infections, making most current treatments ineffective. The authors describe phage therapy as an alternative to conventional treatments, based on antibiotics against uropathogenic bacteria, emphasizing the need for clinical trials to develop and expand the use of phage therapy. Ferriol-González and Domingo-Calap [6] concentrate on the ineffectiveness of current antibacterial treatments, based on antibiotics and disinfectants, to remove bacterial biofilms; biofilm formation, either on a biotic or abiotic surface, currently represents both a major health threat (in infectious diseases) and a source of considerable economic losses. The authors propose the interesting alternative of using phage therapy for biofilm removal; the options available include the application of single lytic phages, phage cocktails, genetically manipulated phages, phage derived enzymes, and the treatment with phages in combination with antibiotics.

Bacterial diseases that attack either agricultural crops, fish, or shellfish not only cause large economic losses, but can even create food shortages, resulting in malnutrition, or even famine, in vulnerable populations. The many years of antibiotic use, and abuse, in either the prevention or treatment of bacterial infections, together with the use of antibiotics in the clinical field, have accelerated the emergence and proliferation of multidrug-resistant bacteria. The review by Sieiro and colleagues [7] focus on the effectiveness of phages in the management of major pathogens that affect both agriculture and aquaculture, with special attention on the scientific and technological aspects that still need further development in order to establish phage therapy as a genuine universal alternative to the use of antibiotics.

This Special Issue also encompasses two review articles that address additional problems resulting from the use of antibiotics, highlighting the need to replace them with alternative treatments, as well as dealing with the possibility of recycling these molecules, in order to recover their activity and use them either individually or as part of combination therapies. The increase in the appearance of resistant bacteria, mentioned above, is not the only adverse effect resulting from the use and abuse of antibiotics. Amadei and Notario [8] report the evidence supporting that exposure to antibiotics not only increases the risk of cancer, but also reduces the effectiveness of various cancer therapies. Accordingly, the authors also discuss alternatives to antibiotics, including phages or enzybiotics. The conclusions arise from the perspective of the new findings on tumor-specific intracellular microbiota and the most recent theories proposed to explain the etiology of cancer. On the other hand, Prudencio et al. [9] concentrate their review on the pharmaceutical applications of ionic liquids (ILs), particularly their potential as antimicrobials and the genuine possibility of using ILs to recycle the classic antibiotics. They demonstrate that ILs can be useful as antimicrobial agents, even against multidrug-resistant strains, either by themselves or in an innovative way, in combination with phage therapy and lysin therapy, creating new strategies to restrain uncontrolled infections.

The articles included here provide an updated platform for discussion and constitute a valuable knowledge reservoir for scientists interested in this field.
